# Final Gleason Score Prediction Using Discriminant Analysis and Support Vector Machine Based on Preoperative Multiparametric MR Imaging of Prostate Cancer at 3T

**DOI:** 10.1155/2014/690787

**Published:** 2014-12-02

**Authors:** Fusun Citak-Er, Metin Vural, Omer Acar, Tarik Esen, Aslihan Onay, Esin Ozturk-Isik

**Affiliations:** ^1^Department of Genetics and Bioengineering, Yeditepe University, İnönü Mah., Kayışdağı Cad, 26 Ağustos Yerleşimi, Ataşehir, 34755 Istanbul, Turkey; ^2^Department of Radiology, VKF American Hospital, 34365 Istanbul, Turkey; ^3^Department of Urology, VKF American Hospital, 34365 Istanbul, Turkey; ^4^School of Medicine, Koç University, 34450 Istanbul, Turkey; ^5^Biomedical Engineering Institute, Boğaziçi University, Rasathane Cad, Kandilli Campus, Kandilli Mah., 34684 Istanbul, Turkey

## Abstract

*Objective.* This study aimed at evaluating linear discriminant analysis (LDA) and support vector machine (SVM) classifiers for estimating final Gleason score preoperatively using multiparametric magnetic resonance imaging (mp-MRI) and clinical parameters. *Materials and Methods.* Thirty-three patients who underwent mp-MRI on a 3T clinical MR scanner and radical prostatectomy were enrolled in this study. The input features for classifiers were age, the presence of a palpable prostate abnormality, prostate specific antigen (PSA) level, index lesion size, and Likert scales of T2 weighted MRI (T2w-MRI), diffusion weighted MRI (DW-MRI), and dynamic contrast enhanced MRI (DCE-MRI) estimated by an experienced radiologist. SVM based recursive feature elimination (SVM-RFE) was used for eliminating features. Principal component analysis (PCA) was applied for data uncorrelation. *Results.* Using a standard PCA before final Gleason score classification resulted in mean sensitivities of 51.19% and 64.37% and mean specificities of 72.71% and 39.90% for LDA and SVM, respectively. Using a Gaussian kernel PCA resulted in mean sensitivities of 86.51% and 87.88% and mean specificities of 63.99% and 56.83% for LDA and SVM, respectively. *Conclusion.* SVM classifier resulted in a slightly higher sensitivity but a lower specificity than LDA method for final Gleason score prediction for prostate cancer for this limited patient population.

## 1. Introduction

Prostate cancer mortality rate has shown an increasing trend in adults over 40 years of age between 1950 and 2008 over the world according to the World Health Organization mortality database [1]. Prostate cancer screening is traditionally based on digital rectal exam (DRE) and prostate specific antigen (PSA) level measurement [2]. Patients with an elevated PSA level or with abnormal findings at DRE are candidates for further transrectal ultrasonography guided prostate biopsy (TRUS-Bx) [3]. However, TRUS-Bx of patients with raised PSA level has a low sensitivity and a high false negative rate (15–30%) due to the limitations of this test [4] and can lead to overdiagnosis and overtreatment of prostate cancers [2].

Multiparametric MRI has recently gained popularity as a tool for prostate cancer diagnosis and staging [5]. Multiparametric prostate MRI examination consists of anatomical and functional MR techniques, such as T1w-MRI, T2w-MRI, DW-MRI, and DCE-MRI, and MR spectroscopic imaging (MRSI). T1w-MRI can be used to detect intraprostatic bleeding, which is a common finding after previous biopsies [2]. T2w-MRI is optimal for depicting the zonal anatomy of the prostate. However, T2w-MRI has significant limitations for depicting cancer in transition zone, because benign prostatic hyperplasia nodules also can have low signal intensity on T2w-MR images depending on the size of stromal component. In addition, many benign conditions such as inflammation, biopsy related hemorrhage, post-radiation therapy fibrosis, and changes after hormone deprivation therapy may be seen as hypointense on T2w-MR images in the peripheral zone. DW-MRI gives information about cellular density through estimating the diffusivity of water molecules, and a decreased free diffusivity of water is seen in prostate carcinomas due to their dense cellularity [2]. Such restriction can be quantitatively evaluated by apparent diffusion coefficient (ADC) maps. In addition, the ADC values have negative correlation with the Gleason score of prostate carcinoma [6].

DCE-MRI is a noninvasive technique that collects information on the tumor angiogenesis. Prostate cancers typically show a steeper wash-in slope, higher peak enhancement, and rapid wash-out compared to healthy prostate tissue [2]. One of the major limitations of DCE-MRI is its nonspecificity. Similarly, prostatitis in the peripheral zone and BPH nodules in the central gland can show earlier and more pronounced enhancement than healthy prostate tissue [7].

Several studies have shown the diagnostic power of multiparametric MRI for prostate cancer. DCE-MRI combined with MRSI was shown to have 93.7% sensitivity and 90.7% specificity for detecting tumor foci in 150 prostate cancer patients [8]. Other studies have shown that combined MRSI and DW-MRI improved prostate cancer detection [9, 10]. Roy et al. reported a specificity of 84.3% for prostate cancer detection before needle biopsy based on DW-MRI, T2w-MRI, and DCE-MRI [11].

Gleason system is commonly used for grading prostate cancer [12]. The tissue obtained either by a biopsy or after a radical prostatectomy is graded from one to five, where a higher grade indicates more aggressiveness. The Gleason score is then calculated as the sum of the most and the second most predominant Gleason grades within the tissue section, and it ranges from two to ten [13]. Predicting the final Gleason score based on preoperative multiparametric MRI through a computer-aided diagnosis (CADx) system has been an ongoing interest. Puech et al. designed a CADx system that successfully determined five-level malignancy suspicion score based on wash-in and wash-out slope values of prostate cancer foci [14]. Sung et al. showed that a CADx system based on nonlinear support vector machine (SVM) outperformed the diagnostic ability of single DCE-MRI parameters and T2w-MRI by a 89% sensitivity and a 89% specificity in the peripheral zone (PZ) [15]. Support vector machine was also used to classify magnetic resonance spectra of prostate in order to assist prostate cancer localization [16]. Poulakis et al. combined PSA, biopsy Gleason score, and magnetic resonance imaging findings for prostate cancer staging using an artificial neural network [17]. Linear discriminant analysis was applied to analyze the power of the 10th percentile and average apparent diffusion coefficient (ADC) values, T2w-MRI signal intensity histogram skewness, and Tofts *K* ^trans⁡^ for the differentiation of prostate cancer from normal tissue [18]. Niaf et al. compared nonlinear SVM, linear discriminant analysis, k-nearest neighbors, and naïve Bayes classifiers to determine an optimal CADx scheme to differentiate malignant tissue from suspicious or nonmalignant tissue at the peripheral zone [19].

In this study, a computer-aided diagnosis system that combined clinical and multiparametric MR findings was developed to predict preoperatively the final Gleason score of prostate cancers. Although various machine learning algorithms have been tested for the prediction and classification of prostate cancer and they mainly differed in the selection of the predictive parameters, according to our knowledge, the 5-point Likert scales of prostate MR images have not been previously evaluated. The main aim was to build a CADx model based on the 5-point Likert scale for multiparametric MRI data classification of prostate cancer in this study. Linear discriminant analysis and support vector machine classifiers were compared for their classification performances after a standard or a Gaussian kernel principal component analysis. Additionally, this work evaluated the contributions of the predictive parameters on prostate cancer malignancy detection by employing an SVM based recursive feature elimination and utilized the kernel trick to enhance the performance of classifiers.

## 2. Materials and Methods

### 2.1. Subjects

Thirty-three prostate cancer patients (mean (±std.) age = 61.5 ± 5.9, range = 46–71), who subsequently underwent radical prostatectomy, were included in this study. The institutional research committee approved this retrospective study. Routine clinical examination included digital rectal examination to detect the presence of a palpable prostate abnormality and serum PSA level measurement. Index lesion size was measured based on mp-MRI, and Gleason scores were determined based on the pathologic analysis of radical prostatectomy specimen.

### 2.2. MRI Data Acquisition and Feature Extraction

All patients were scanned on a 3T clinical MRI scanner (Magnetom Skyra, Siemens AG, Erlangen, Germany), using sixteen-channel phased array body coil. Before MR imaging, all patients were injected intramuscularly 20 mg of butylscopolamine (Buscopan; Boehringer, Germany) to suppress bowel peristalsis. Multiparametric MR imaging protocol included 2D T2w-MRI, DW-MRI, and DCE-MRI. T2w-MRI was obtained in three orthogonal planes using T2w turbo spin echo (TSE) sequence. DW-MRI was obtained in axial orientation using a spin echo-echo planar imaging (SE-EPI) sequence with six *b* values (0, 50, 250, 500, 800, and 1000 s/mm²) and computed DW MR images were generated for *b*-values of 1200 and 1500 s/mm^2^. Restriction of diffusion was quantified by the apparent diffusion coefficient (ADC) value. DCE-MRI was obtained using a fast 3D T1-weighted (T1-VIBE) gradient echo sequence in axial orientation. Each volumetric acquisition of the DCE sequence had an acquisition time of 7 seconds. A total of 35 contrast-enhanced acquisitions were performed. Perfusion curves were generated by using the image processing tools of an MRI CAD system (Dynacad; Invivo, Birmingham, MI). All MR images were interpreted by an experienced radiologist (14 years of experience in abdominal MRI and 4 years in prostate mp-MRI), who was informed about the clinical findings of the patients. Low signal on T2w-MRI, low apparent diffusion coefficient (ADC) values (<1000), high signal intensity on high *b* value imaging (≥800), and types 2-3 enhancement curve after contrast administration were interpreted as the main radiological criteria for an underlying prostate malignancy. ADC images were generated on the MRI scanner console. A Likert score was designated for T2w-MRI, DW-MRI, and DCE-MRI within each region of interest according to a 5-point scale (i.e., the presence of clinically significant cancer is as follows: 1 = “extremely unlikely”, 2 = “unlikely”, 3 = “equivocal”, 4 = “likely”, and 5 = “extremely likely”) based on ESUR guidelines [20].

The age of the patient, the presence or absence of a palpable prostate abnormality from digital rectal examination findings, PSA level, index lesion size based on mp-MRI, and Likert scales of T2w-MRI, DW-MRI, and DCE-MRI were used as the predictors. In this study, rather than all tumor foci, the index lesions have been evaluated. The index lesion was considered to be the largest lesion with a high Gleason score. In our study dataset, largest lesions had higher Gleason scores than other tumor foci. Gleason scores 3 + 3 (*n* = 3) and 3 + 4 (*n* = 17) were defined as low-grade (*L*) [21], whereas Gleason scores 4 + 3 (*n* = 7), 4 + 4 (*n* = 2), 4 + 5 (*n* = 2), and 5 + 4 (*n* = 2) were defined as high-grade (*H*) in this small patient population. The age of the patients was mapped into binary values, where patients who were older than 65 [22] were considered as old (1) and otherwise as young (0).

A computer-aided diagnosis (CADx) system was designed for interpreting multiparametric prostate cancer MRI data. Feature elimination using support vector machine based recursive feature elimination, data uncorrelation using principal component analysis (PCA) with or without a Gaussian kernel, classification, and evaluation were the major stages of this CADx. The performance of two classification algorithms, which were the linear discriminant analysis and linear SVM, was assessed for predicting the final Gleason score of radical prostatectomy specimen based on the chosen seven clinical and radiological predictors.

### 2.3. Feature Elimination

Support vector machine based recursive feature elimination [23] was used to eliminate the least important feature before subsequent analytical operations for assessing the contribution of the features. As a first step, a support vector machine was trained using the dataset defined as

(1) $$\begin{array}{c} S={\left\{\left(x_{i},y_{i}\right)\right\}}_{i=1,n},\;x_{i}\in R^{f},\;\;y_{i}\in \left\{-1,+1\right\}, \end{array}$$

where *f* is the number of features, *n* is the number of samples, *x* are the feature vectors of size *f*, and *y* are the outputs of the corresponding feature vector *x*. The possible values for *y* were (−1) and (+1), which indicated the low and high Gleason score groups, respectively.

As a second step of the feature elimination, the Lagrange multipliers are used to calculate the scores of the features individually. The score of a feature, *j*, is the square of the sum of the product of *α*, *y* and the value of that feature for all support vectors, formulated as

(2) $$\begin{array}{c} r_{j}={\left(\overset{k}{\underset{i=1}{\sum }}\alpha_{i}y_{i}x_{i,j}\right)}^{2}, \end{array}$$

where *α*_*i*_, *y*_*i*_, and *x*_*i*,*j*_ are the Lagrange multiplier, the output, and the value of the *j*th feature of the *k* number of support vectors, respectively. Finally, the feature having the minimum score can be eliminated. In this study, the least important feature was eliminated at each step.

### 2.4. Data Uncorrelation

After feature elimination, standard and kernel principal component analysis (kernel PCA) was used to uncorrelate the data [24]. Standard principal component analysis aims to find a new coordinate system, which is composed of a set of orthogonal vectors, called the “principal components” (PC). Kernel PCA is the nonlinear form of PCA that nonlinearly maps the dataset to a higher dimensional feature space via kernel trick, which uses kernel matrix instead of the covariance matrix unlike standard PCA [25]. Since standard PCA produces linear feature space, it is not suitable for complex data distributions. Kernel PCA outperforms standard PCA in most cases [26].

In this study, Gaussian kernel PCA was implemented in MATLAB using kernel PCA and pre-image reconstruction software [27]. The data was placed in an *n* × *f* data matrix, where the rows represented *n* number of samples and columns were *f* number of features. Instead of calculating the covariance matrix, the data was transformed into an *n* × *n* dimensional high feature space with the Gaussian kernel function as

(3) $$\begin{array}{c} K\left(x\right)=\exp\left(\frac{-{\left\|x\right\|}^{2}}{2\sigma^{2}}\right), \end{array}$$

where *x* is the dataset and *σ* is a performance regularization constant of the kernel. Then, standard PCA was implicitly applied to the kernel matrix. Therefore, the original dataset was projected into the coordinate system of the eigenvectors, which is a nonlinear representation of the original dataset. The scree graph [24] was used to determine the cut-off point for the number of principal components to represent the data.

### 2.5. Classification Based on Discriminant Analysis

Linear discriminant analysis searches for a vector, *w* ^*T*^, called a discriminant transformation function that results in the minimum intraclass and the maximum interclass distances when a data is projected onto it [28]. Given a dataset *X* as

(4) X=xi,yii=1,n, xi∈Rf, yi∈0,+1,hhhhhhhhhhhihhhhyi=v  then  yi∈Cv,

a projection *z* of *x* onto the vector *w* can be defined as

(5) $$\begin{array}{c} z=w^{T}x. \end{array}$$

LDA searches for such a discriminant transformation function that separates the means of the classes as much as possible after the projection, while keeping scatters of the projection for each class as small as possible [28]. In this study, LDA, which was implemented in MATLAB, was used as one of the classification methods.

### 2.6. Classification Based on Support Vector Machines

Support vector machine is a supervised machine learning technique used for classification and regression analysis [29]. SVM algorithm tries to construct an optimal separating hyperplane that maximizes the margin, where the margin is the largest distance to the nearest training data point of any class. In this study, support vector machine with linear kernel was implemented using Statistics Toolbox 8.1 (R2013a) of MATLAB (The Mathworks Inc., Natick, MA). A linear kernel, *K*, maps the original data with the kernel function as

(6) $$\begin{array}{c} K\left(x\right)=\left(x\cdot x^{\prime }+c\right), \end{array}$$

where *x* is the data and *c* is an optional constant.

### 2.7. Performance Evaluation

Ten-fold crossvalidation method was used to compare the performances of the classification models. The sensitivity and specificity values were calculated for each test fold on the trained model of the other nine folds. The performance of each iteration was calculated as the average of the performance values of these ten folds. This procedure was repeated for thirty times. The accuracy, sensitivity, and specificity values of the two kinds of PCA were compared using a Mann-Whitney rank sum test to detect pairwise statistically significant performance differences between classifiers. A multiple comparison correction was applied using Bonferroni correction, and a *P* value of less than 0.05/24 = 0.002 was considered statistically significant.

## 3. Results

**Figure 1:**
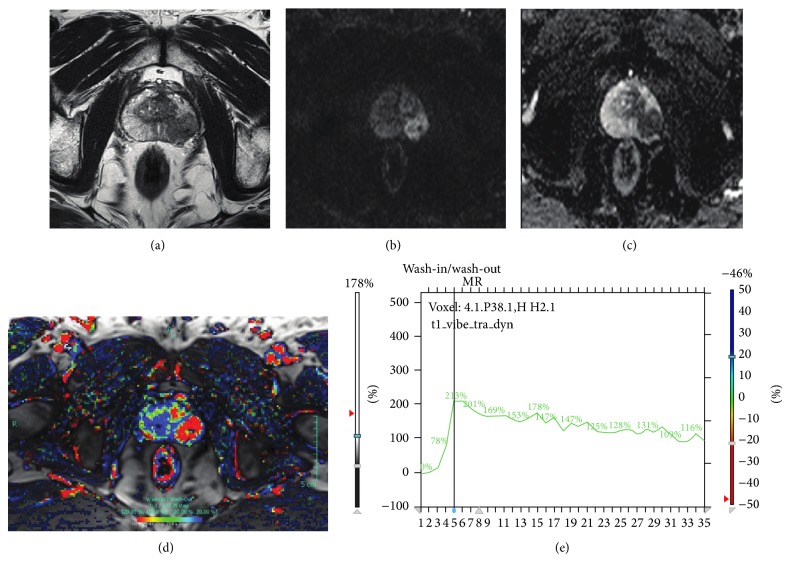
Multiparametric MRI data of a 67-years old male patient diagnosed with prostate cancer. T2w-MRI (a), DW-MRI (*b* = 1000) (b), ADC (c), DCE-MRI wash-in/wash-out map (d), and DCE-MRI time curve (e).

Figure 1 shows the multiparametric MRI data of a 67-years old patient diagnosed with a prostate cancer. There was a signal intensity wash-out in the DCE-MRI data (Figure 1(d)), low T2 signal intensity (Figure 1(a)), and low ADC (Figure 1(c)).

Table 1 shows the number of subjects for age and DRE and the mean and standard deviation values for the other five features for the low and high Gleason score groups. Younger patients tended to have more low-grade lesions. There were also less DRE findings in the low-grade group. The high Gleason score patients had a higher PSA, index lesion size, and Likert scales of DW-MRI and DCE-MRI.

Support vector machine based feature elimination was repeated three times, and the feature having the least score was eliminated from the analysis at each iteration.  Table 2 shows the scores of features at each iteration, where the features having the least score within each iteration are marked as bold. Digital rectal exam findings, age, and the lesion size were eliminated from the classification after three iterations.

**Figure 2:**
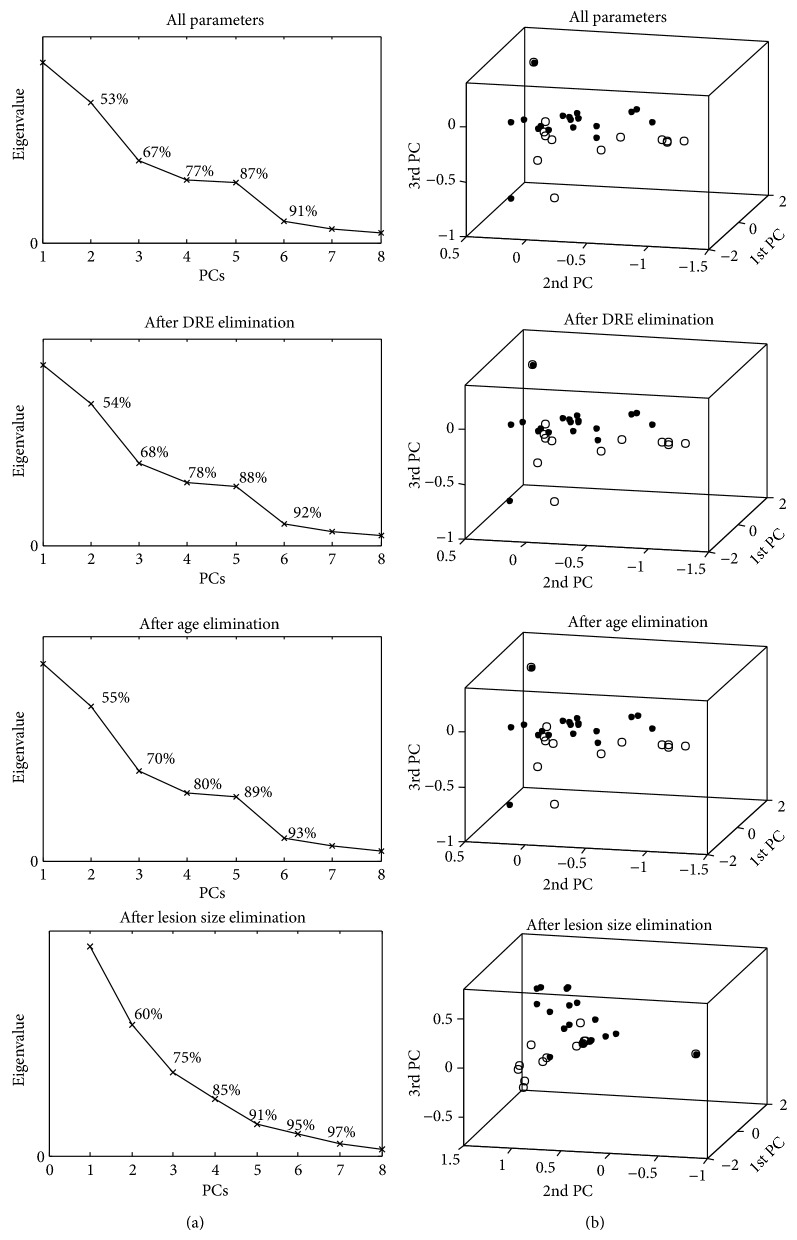
The results of data uncorrelation via Gaussian kernel PCA after each iteration of SVM-RFE. The scree plots of the eigenvalues versus principal components (PCs) (a) and the distribution of the first three principal components at each data point (b) are given. The high-grade data are labeled as circles, while the low-grade data are labeled as dots.

Then, a standard or a Gaussian kernel PCA was applied to uncorrelate the data after each iteration of the SVM-RFE. The sigma (*σ*) parameter of Gaussian kernel and the size of the remaining dimension were considered separately at each iteration.  Figure 2 shows the results of data uncorrelation via Gaussian kernel PCA after each iteration of SVM-RFE. The scree plots of the eigenvalues versus the principal components with the corresponding cumulative percent variances and the first three principal components of the uncorrelated data are shown. The principal component located at the elbow of the scree plot was selected as the cut-off point for the total number of principal components. A cut-off point that keeps 80% to 90% of the data variance was selected [30].

Table 3 shows the averages and confidence intervals of thirty iterations of the accuracies, sensitivities, and specificities for the four combinations of Gaussian kernel PCA and standard PCA with LDA classifier and SVM classifier. All accuracy and sensitivity values were statistically significantly different between the Gaussian kernel PCA and standard PCA based on a Mann-Whitney rank sum test (*α* < 0.002). The specificities of LDA methods were not statistically significantly different when applied after two kinds of PCA methods. The performance measurements of LDA and SVM methods were statistically significantly different when standard PCA and SVM-RFE were applied (*α* < 0.002). Gaussian kernel PCA significantly outperformed standard PCA in all cases. Feature elimination with SVM-RFE increased the classification performance of SVM in terms of accuracy and sensitivity.

## 4. Discussion

Multiparametric MRI has been reported to have diagnostic value for prostate cancer. The value of ADC has been found to be strongly correlated with the aggressiveness of prostate cancer [6, 31, 32] and the tumor growth rate [33]. It was reported that the T2^*^ values of the cancerous prostatic regions were significantly lower than those of the benign prostatic regions [34]. It was proposed that quantitative diffusion tensor imaging (DTI) analysis can be used to discriminate prostate cancer from normal tissue [35]. Transition zone entropy of T2 and T1 weighted images obtained by application of spatial filters was proposed as a new promising diagnostic feature for the development of mp-MRI based CADx systems [36]. Vos et al. concluded that the combinational use of DW-MRI and T2w-MRI is sufficient for the assessment of prostate cancer aggressiveness in the peripheral zone, while DCE-MRI and MRSI should be additionally considered for discrimination of indolent and high-grade prostate cancers [37].

Many subsets of mp-MRI features have been proposed as the candidate predictors for a computer-aided prostate cancer diagnosis system in the literature. Grey-level histogram of T2w-MRI, ADC value, and semi-quantitative features extracted from DCE curves were used as the features of a prostate cancer CADx system, where sequential forward selection (SFS) feature selection algorithm was employed to determine the best combination of these features [38]. Several combinations of features extracted from DTI [39] and DCE-MRI [14, 39–41] were used for detection and grading of prostate cancer. A logistic regression model was fitted using ADC, elevated choline peaks in MR spectroscopy, increased perfusion, and malignant wash-out parameters for prostate cancer detection [42]. The ratios of sum of total choline, spermine, and creatine (CSC) to citrate (CSC/Cit) and total choline to citrate (tCho/Cit) were used as predictors of two different CADx models to assess the aggressiveness of prostate cancer, with resultant sensitivities of 87% and 81%, respectively [43]. Support vector machines were used to build an image-based computer-aided diagnosis system, which creates a cancer probability map of prostate cancer based on DTI and DCE-MRI [39]. The combination of 10th percentile of ADC, average ADC, and T2-weighted skewness was used to build a CADx system for differentiating normal and tumor tissues in the prostate [18].

On the other hand, mp-MRI approach has some limitations. Some of these MRI modalities might not be available for each patient. Some solutions were proposed to estimate missing DTI and DCE parameters [44]. Also, T2w-MR imaging has been reported as being difficult to incorporate into a CAD system due to its sensitivity to patient motion [45]. To our knowledge, the 5-point Likert scales of prostate MR images have not been previously used as predictors of a CADx system for the pre-operative prediction of final Gleason score. The Likert scale encompasses the mp-MRI information in a more standardized way, which might result in less variability between different sites. Furthermore, clinical findings were also incorporated in this study.

The main limitation of this study was the small patient data size. Although the study has been ongoing for the past two years, only thirty-three patients, who later underwent a radical prostatectomy, have been successfully recruited. The main reason for low specificity was the lower number of high-grade data (*n* = 13) than the low-grade (*n* = 20). As a result, even a small number of false positives resulted in a noticeable loss in specificity.

Additionally, a kernel PCA was employed to extract nonlinear relationship between predictors. The most suitable kernel was searched to improve the efficiency of PCA, and the best results were achieved when a Gaussian kernel was employed. For this small dataset, Gaussian kernel PCA worked noticeably better than the standard PCA. However, the computational complexity of kernel PCA is higher than the standard PCA. Kernel PCA processes an *n* × *n* kernel matrix, where *n* is the size of the training vector, while standard PCA works on an *f* × *f* covariance matrix, where *f* is the number of features of the training vector. Several enhancements of kernel PCA have been proposed in the literature to solve this issue, which will be considered in the future studies as the size of the data increases [26]. More suitable kernels for PCA should also be investigated in future studies.

Selecting the best subset of features in a large hypothesis space defined by all possible combinations of features is a well-known issue in machine learning. The main advantage of the feature elimination is to avoid overfitting, which means a perfect fit of the decision function for the training dataset, but a failed fit for the subsequent test datasets. In this study, SVM-RFE was used to eliminate redundant features for a better classification and to avoid overfitting. The classifiers often perform better after feature elimination [28]. However, there is a trade-off between feature elimination and classification. If the classifier is sufficiently strong, then feature elimination might not be necessary. In this study, mean sensitivities of 81.59%, 83.38%, 55.72%, and 58.16% and mean specificities of 70.09%, 68.36%, 67.55%, and 47.87% were attained for LDA with Gaussian kernel PCA, SVM with Gaussian kernel PCA, LDA with standard PCA, and SVM with standard PCA, respectively, when feature elimination was not performed. The mean sensitivities of the classifiers with Gaussian kernel PCA were statistically significantly higher while the mean specificities of them were slightly lower when all feature elimination iterations were applied. It was observed that the specificity of SVM was more affected from feature elimination than LDA; however, it was essential to apply feature elimination for a higher sensitivity and for assessing the contribution of each feature for classification.

## 5. Conclusion

The application of a Gaussian kernel PCA increased the performances of both classification models for an accurate prediction of final Gleason score based on clinical findings and preoperative multiparametric MR imaging for this limited patient population, while exploiting the complicated relationship between features. Linear discriminant analysis provided a slightly higher specificity than the SVM method, which can be related to the small intraclass distance. It was observed that mp-MRI features were more important than clinical features based on SVM-RFE scores, and applying feature elimination procedure increased the classification performances of the models. One novelty of this study is modeling a prediction system based on Likert scales of mp-MRI rather than a set of mp-MRI features, which might be a more standardized way of assessing mp-MRI data. Future studies will investigate the role of Likert scales of preoperative MR imaging parameters in final Gleason score prediction in a larger patient population. The analytical results of a CADx system based on multiparametric MRI might help radiologists in clinical decision making.

## Figures and Tables

**Table 1:** The number of subjects for the two binary features (age and DRE), and the mean (±std.) values for the other five features grouped by low and high Gleason score patients.

	Low-grade (*n* = 20)		High-grade (*n* = 13)	
Age	(<65)	16	(<65)	7
	(≥65)	4	(≥65)	6
DRE	No	17	No	8
	Yes	3	Yes	5
PSA	8.42 ± 12.92		9.91 ± 9.35	
Index lesion size	1.64 ± 1.78		2.23 ± 1.64	
Likert scale of T2w-MRI	3.90 ± 0.72		4.00 ± 0.71	
Likert scale of DW-MRI	4.55 ± 0.60		4.69 ± 0.48	
Likert scale of DCE-MRI	3.55 ± 1.47		4.62 ± 0.51	

**Table 2:** The scores calculated by SVM-RFE in three iterations.

	1st iteration	2nd iteration	3rd iteration
Age	32.94	**35.2**	**—**
DRE	**21.65**	**—**	—
PSA	18451.23	18807.95	18992.86
The size of lesion	881.93	900.45	**930.07**
T2w-MRI Likert scale	2899.26	3029.82	3179.27
DW-MRI Likert scale	4055.23	4232.81	4423.32
DCE-MRI Likert scale	3857.99	4027.87	4242.97

^*^The eliminated features are marked as bold, which are then not included in the subsequent iterations.

**Table 3:** The averages and confidence intervals of accuracy, sensitivity, and specificity values for the combinations of two kinds of classifiers and two kinds of PCA averaged after thirty iterations.

			Accuracy [CI] (%)		Sensitivity [CI] (%)		Specificity [CI] (%)	
All parameters	SVM	Kernel PCA	76.83 [75.77–77.90]	∗	83.38 [81.77–85.00]	∗	68.36 [65.88–70.84]	∗
		Standard PCA	52.03 [50.52–53.53]		58.16 [55.94–60.38]		47.87 [43.93–51.8]	
	LDA	Kernel-PCA	76.56 [75.42–77.70]	∗	81.59 [79.87–83.31]	∗	70.09 [67.66–72.52]
		Standard PCA	60.03 [58.88–61.17]		55.72 [53.98–57.45]		67.55 [64.19–70.91]

DRE eliminated	SVM	Kernel PCA	76.17 [75.12–77.21]	∗	84.59 [83.22–85.96]	∗	66.07 [63.67–68.46]	∗
		Standard PCA	51.94 [50.17–53.72]		58.24 [55.92–60.55]		46.11 [41.70–50.53]	
	LDA	Kernel PCA	75.36 [74.01–76.71]	∗	82.59 [80.84–84.34]	∗	66.27 [63.75–68.79]
		Standard PCA	60.11 [59.09–61.13]		55.28 [53.61–56.94]		67.14 [63.83–70.46]

Age eliminated	SVM	Kernel PCA	75.97 [75.07–76.88]	∗	85.12 [83.73–86.51]	∗	64.15 [61.57–66.72]	∗
		Standard PCA	56.03 [53.97–58.09]		67.85 [65.64–70.07]		40.55 [35.62–45.49]	
	LDA	Kernel PCA	76.33 [75.10–77.56]	∗	83.95 [82.10–85.80]	∗	66.48 [63.27–69.69]
		Standard PCA	57.50 [56.11–58.89]		51.81 [49.34–54.28]		65.01 [62.26–67.77]

Index Lesion Size Eliminated	SVM	Kernel PCA	75.31 [74.19–76.42]	∗	87.88 [86.31–89.45]	∗	56.83 [53.94–59.72]	∗
		Standard PCA	53.64 [52.52–54.76]		64.37 [62.52–66.22]		39.90 [36.66–43.15]	
	LDA	Kernel PCA	76.75 [75.64–77.86]	∗	86.51 [84.94–88.08]	∗	64.00 [61.22–66.77]	∗
		Standard PCA	59.69 [58.36–61.03]		51.20 [48.89–53.50]		72.71 [69.60–75.81]	

^*^Statistically significantly different (*α* < 0.05/24).
